# Supplementary material for: Targeting with Structural Analogs of Natural Products the Purine Salvage Pathway in Leishmania (Leishmania) infantum by Computer-Aided Drug-Design Approaches
Source: Trop Med Infect Dis. 2024 Feb 3;9(2):41. doi: 10.3390/tropicalmed9020041 (PMC10891554; doi:10.3390/tropicalmed9020041)
Supplement: Supplementary file 1 [file tropicalmed-09-00041-s001.zip › tropicalmed-2771539-supplementary-proof done.pdf]

Supplementary Materials: Targeting with structural analogs of natural products the purine salvage pathway in *Leishmania (Leishmania) infantum* by computer-aided drug design approaches

Haruna Luz Barazorda-Ccahuana[1], Eymi Gladys Cárcamo-Rodriguez[1,2], Angela Emperatriz Centeno-Lopez[1,2], Alexsandro Sobreira Galdino[3], Ricardo Andrez Machado-de-Ávila[4], Rodolfo Cordeiro Giunchetti[5,6], Eduardo Antonio Ferraz Coelho[7,8], Miguel Angel Chávez-Fumagalli[1]*

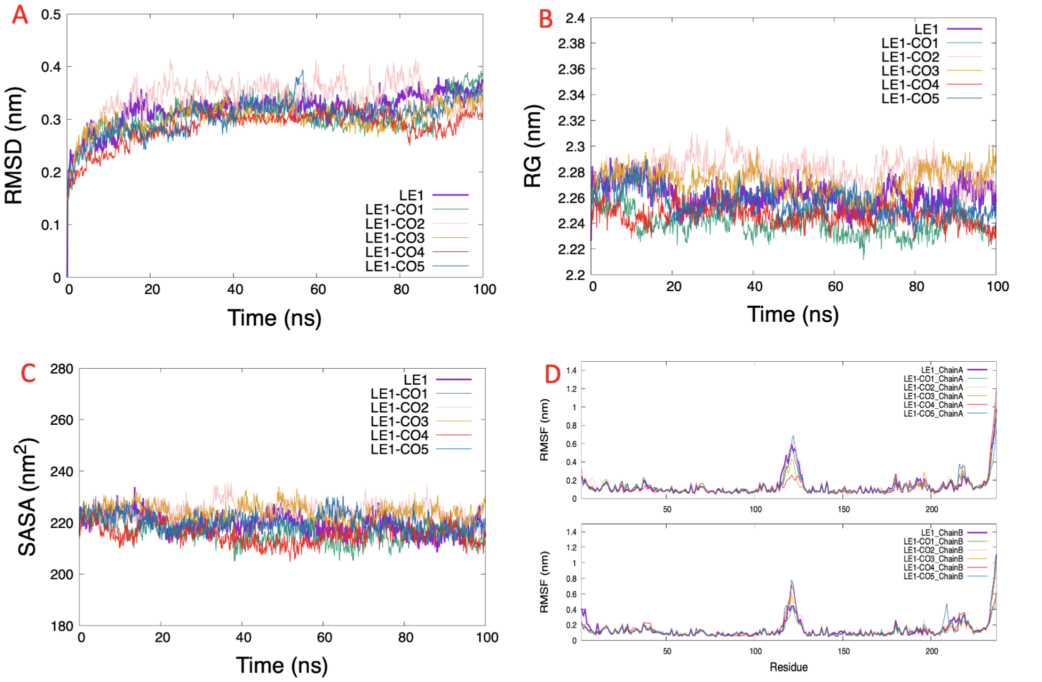

Figure S1. RMSD, SASA, and RG analysis of Adenine phosphoribosyltransferase. (A) RMSD graph. (B) RG graph. (C) SASA graph. (D) RMSF graph.

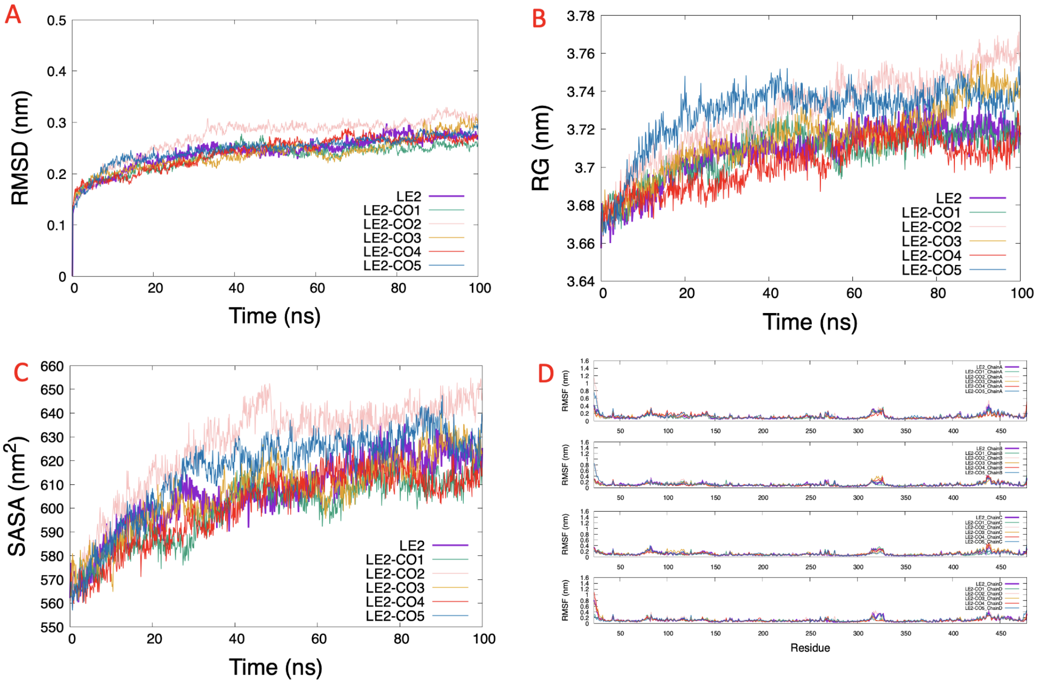

Figure S2. RMSD, SASA, and RG analysis of Adenylosuccinate lyase. (A) RMSD graph. (B) RG graph. (C) SASA graph. (D) RMSF graph.

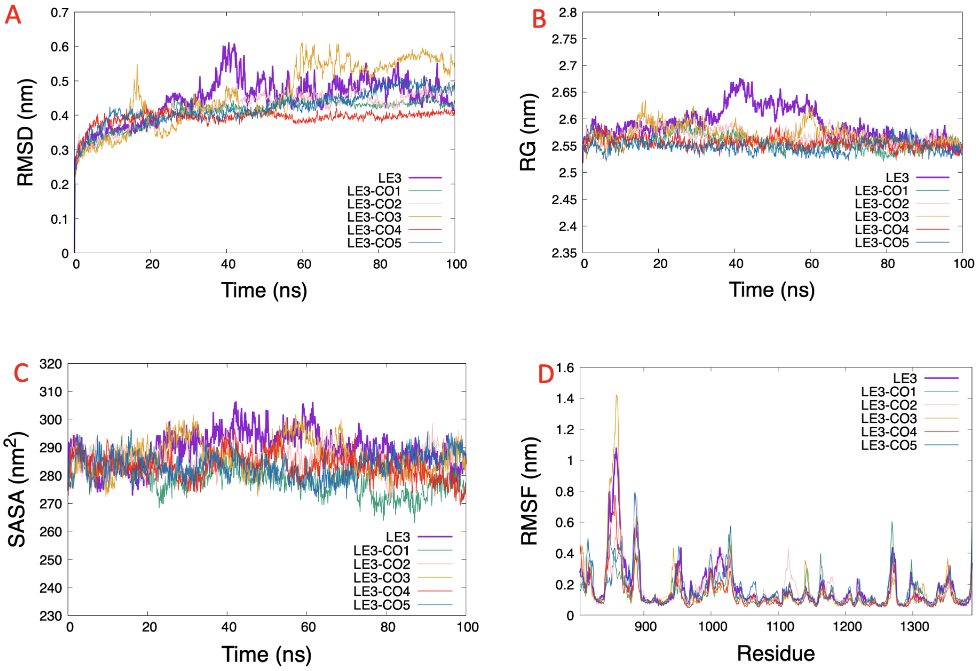

Figure S3. RMSD, SASA, and RG analysis of Putative AMP deaminase. (A) RMSD graph. (B) RG graph. (C) SASA graph. (D) RMSF graph.

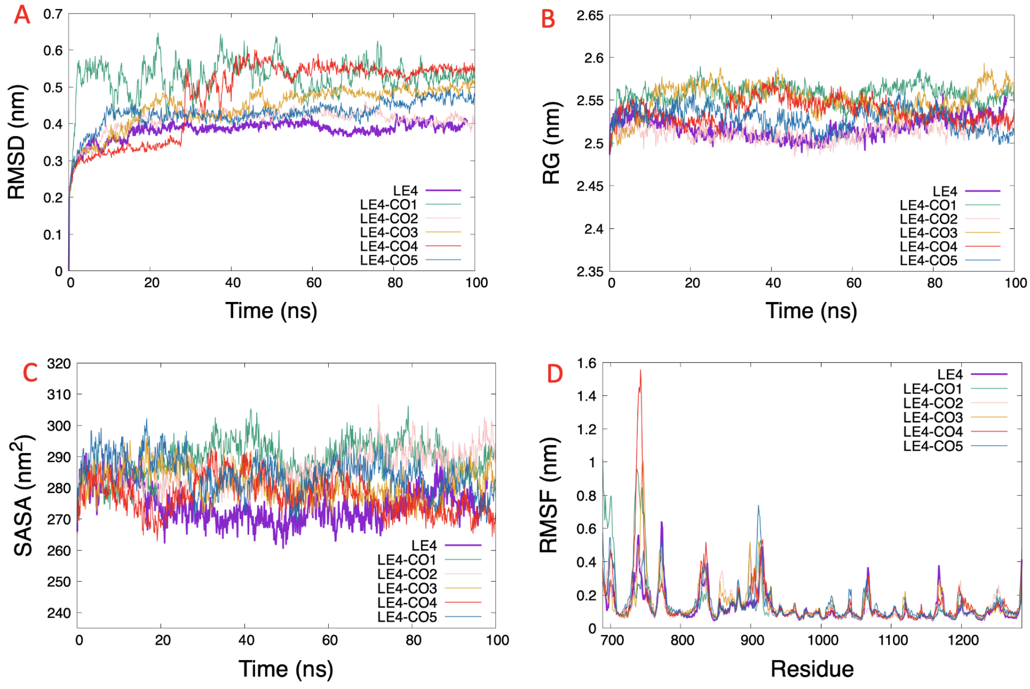

Figure S4. RMSD, SASA, and RG analysis of AMP deaminase. (A) RMSD graph. (B) RG graph. (C) SASA graph. (D) RMSF graph.

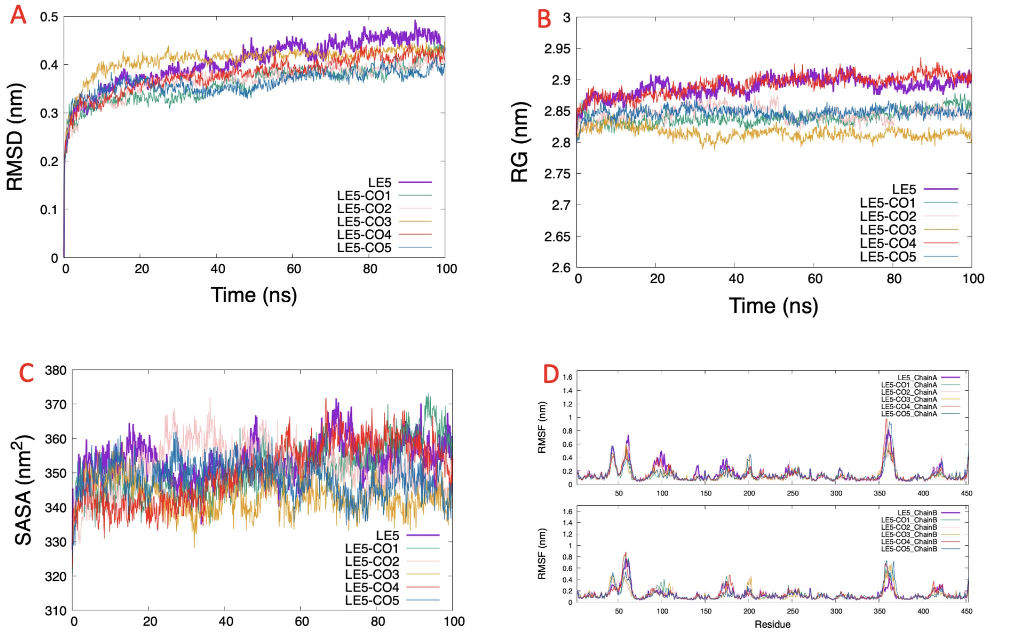

Figure S5. RMSD, SASA, and RG analysis of Guanine deaminase. (A) RMSD graph. (B) RG graph. (C) SASA graph. (D) RMSF graph.

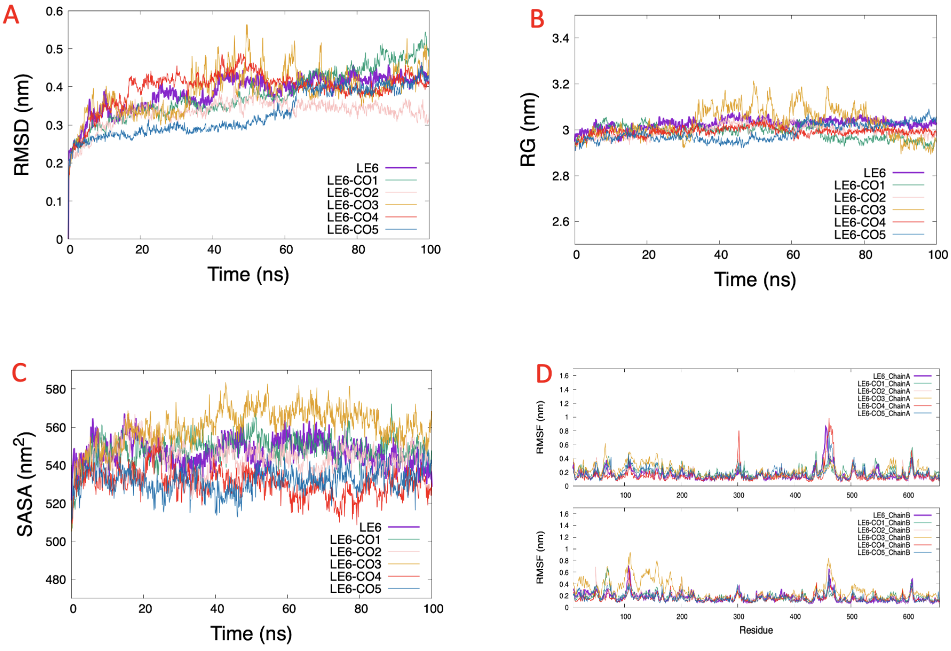

Figure S6. RMSD, SASA, and RG analysis of GMP synthase. (A) RMSD graph. (B) RG graph. (C) SASA graph. (D) RMSF graph.

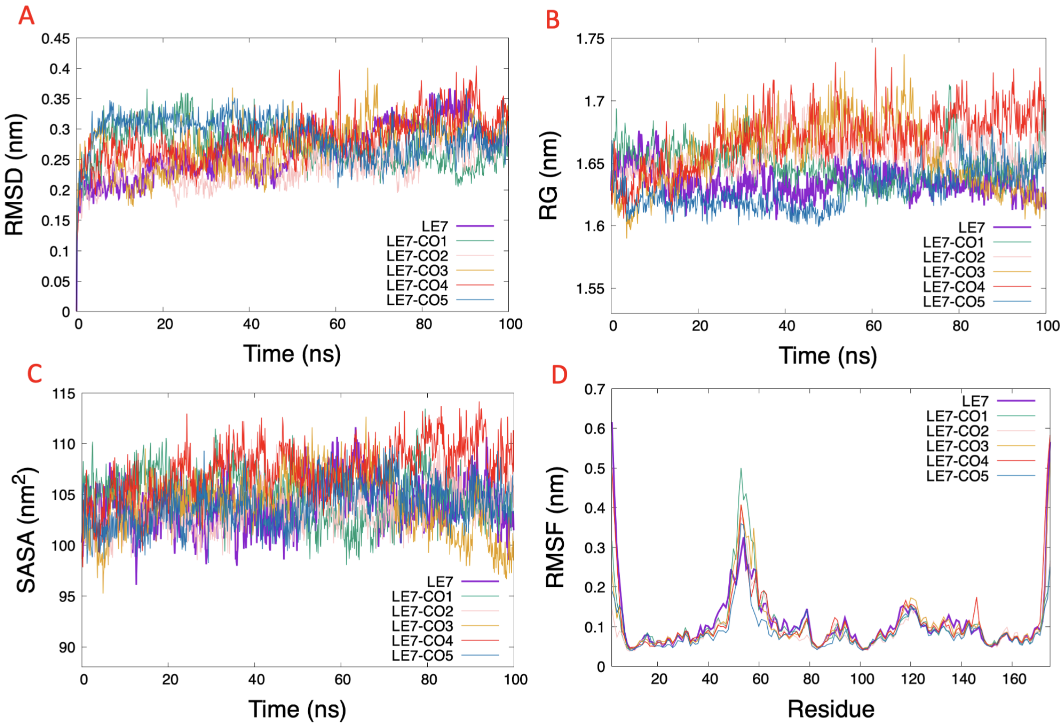

Figure S7. RMSD, SASA, and RG analysis of Adenylate kinase isoenzyme. (A) RMSD graph. (B) RG graph. (C) SASA graph. (D) RMSF graph.

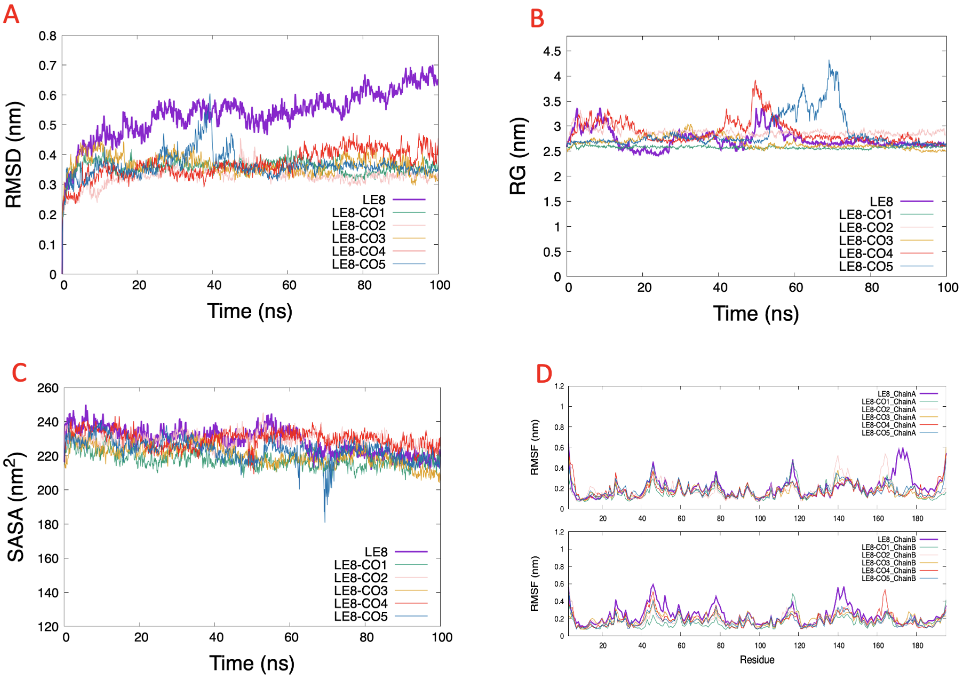

Figure S8. RMSD, SASA, and RG analysis of Guanylate kinase-like protein. (A) RMSD graph. (B) RG graph. (C) SASA graph. (D) RMSF graph.

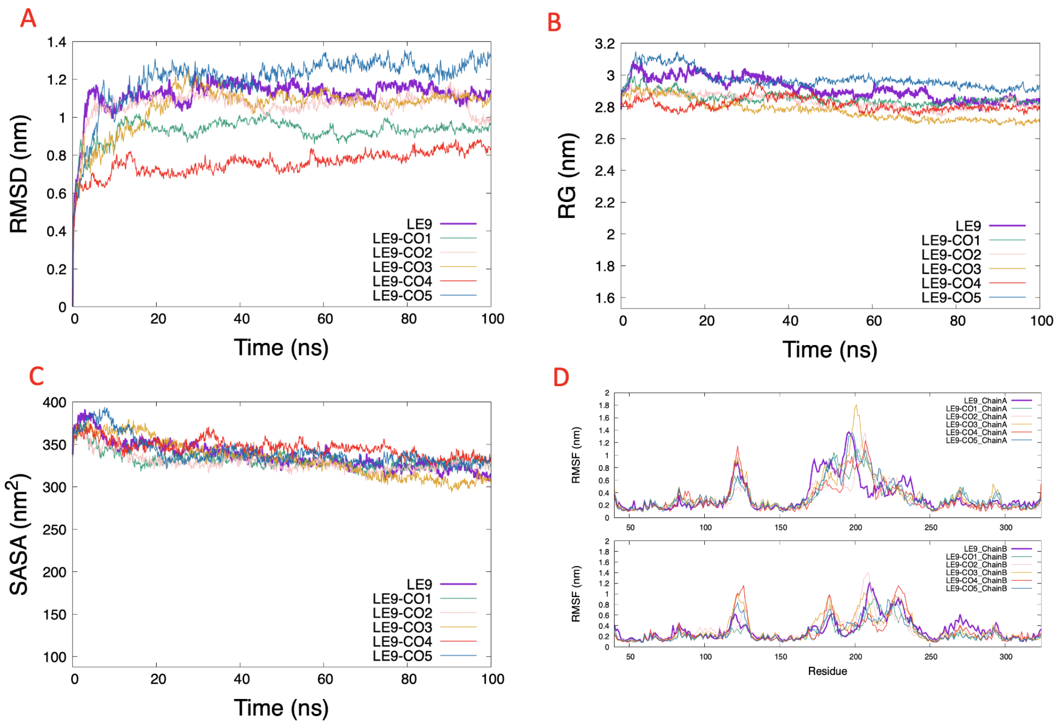

Figure S9. RMSD, SASA, and RG analysis of Guanylate kinase-like protein. (A) RMSD graph. (B) RG graph. (C) SASA graph. (D) RMSF graph.

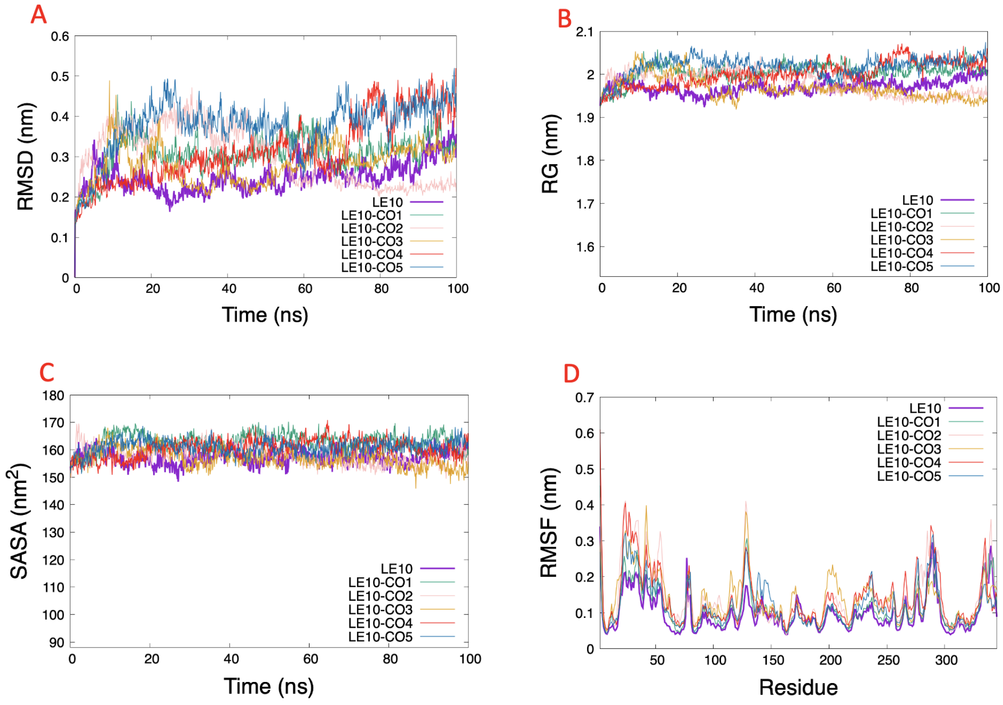

Figure S10. RMSD, SASA, and RG analysis of Adenosine kinase. (A) RMSD graph. (B) RG graph. (C) SASA graph. (D) RMSF graph.

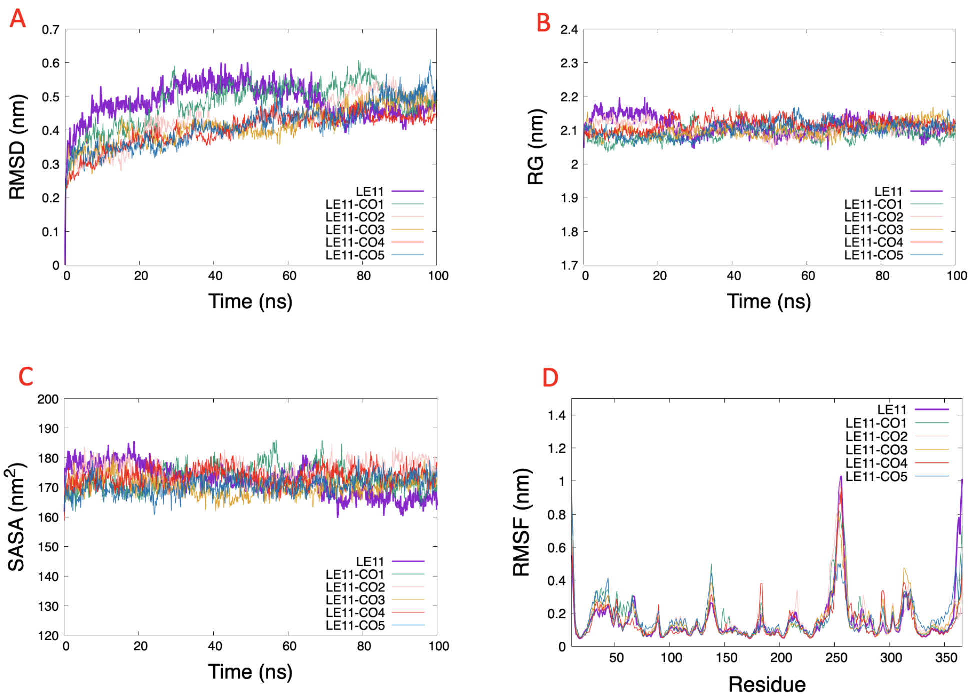

Figure S11. RMSD, SASA, and RG analysis of Adenosine kinase. (A) RMSD graph. (B) RG graph. (C) SASA graph. (D) RMSF graph.

Table S1. Quality values of the templates used in homology modeling through SwissModel server.

Target	Template	Seq Identity	Oligo-state	QSQE	Found by	Method	Resolution	Seq Similarity	Range	Coverage	Description
Adenine phosphoribosyltransferase	1qb7.1.A	100	homo-dimer	0.9	BLAST	X-ray	1.50Å	0.61	2 - 237	1	Adenine Phosphoribosyltransferase
Adenylosuccinate lyase	4efc.1.A	66.74	homo-tetramer	0.91	HHblits	X-ray	2.00Å	0.51	23 - 478	0.96	Adenylosuccinate lyase
Putative AMP deaminase	A4I876.1.A	100	monomer	-	AFDB search	AlphaFold v2	-	0.62	1 - 1612	1	Putative AMP deaminase
AMP deaminase	A4IC17.1.A	100	monomer	-	AFDB search	AlphaFold v2	-	0.62	1 - 1472	1	AMP deaminase
Guanine deaminase	A4I4E1.1.A	100	monomer	-	AFDB search	AlphaFold v2	-	0.61	1 - 454	1	Guanine deaminase
GMP synthase	E9AGZ1.1.A	100	monomer	-	AFDB search	AlphaFold v2	-	0.61	1 - 656	1	GMP synthase (glutamine-hydrolyzing)
Adenylate kinase isoenzyme 6	A4I5L5.1.A	100	monomer	-	AFDB search	AlphaFold v2	-	0.61	1 - 180	1	Adenylate kinase isoenzyme 6 homolog
Guanylate kinase	A4I8X4.1.A	100	monomer	-	AFDB search	AlphaFold v2	-	0.61	1 - 203	1	guanylate kinase
Guanylate kinase-like	A4IDK0.1.A	100	monomer	-	AFDB search	AlphaFold v2	-	0.61	1 - 329	1	Guanylate kinase-like protein
Adenosine kinase	A4I5C0.1.A	100	monomer	-	AFDB search	AlphaFold v2	-	0.61	1 - 345	1	Adenosine kinase
Adenosine kinase	A4IAC6.1.A	100	monomer	-	AFDB search	AlphaFold v2	-	0.61	1 - 388	1	Adenosine kinase

Table S2. RMSD, RG, and SASA average values of eleven targets over 100 ns of MD simulations.

System	RMSD (nm)	RG(nm)	SASA(nm^2)
LE1	0.32±0.03	2.26±0.01	219.33±3.88
LE1-CO1	0.30±0.04	2.24±0.01	216.14±3.98
LE1-CO2	0.34±0.04	2.28±0.01	223.98±3.61
LE1-CO3	0.30±0.03	2.27±0.01	223.84±3.28
LE1-CO4	0.28±0.03	2.25±0.01	215.63±3.95
LE1-CO5	0.30±0.04	2.26±0.01	220.18±3.48
LE2	0.24±0.03	3.71±0.01	606.46±15.06
LE2-CO1	0.23±0.03	3.71±0.01	599.43±13.79
LE2-CO2	0.27±0.04	3.73±0.02	626.49±20.15
LE2-CO3	0.24±0.03	3.72±0.02	606.64±15.38
LE2-CO4	0.24±0.03	3.70±0.01	600.56±15.66
LE2-CO5	0.25±0.03	3.73±0.02	615.86±18.40
LE3	0.45±0.06	2.59±0.03	289.41±6.03
LE3-CO1	0.41±0.04	2.56±0.01	278.67±5.23
LE3-CO2	0.42±0.05	2.57±0.01	285.27±4.17
LE3-CO3	0.46±0.09	2.57±0.02	286.61±5.77
LE3-CO4	0.39±0.02	2.55±0.01	283.35±4.61
LE3-CO5	0.42±0.05	2.55±0.01	283.56±4.56
LE4	0.38±0.03	2.52±0.01	274.58±5.70
LE4-CO1	0.53±0.05	2.56±0.01	289.16±5.79
LE4-CO2	0.41±0.03	2.51±0.01	285.96±5.56
LE4-CO3	0.45±0.06	2.55±0.02	281.28±5.06
LE4-CO4	0.48±0.10	2.54±0.01	277.42±5.80
LE4-CO5	0.43±0.04	2.53±0.01	284.31±5.85
LE5	0.41±0.05	2.89±0.01	353.52±5.78
LE5-CO1	0.36±0.04	2.84±0.01	350.91±7.54
LE5-CO2	0.37±0.03	2.85±0.01	351.17±6.89
LE5-CO3	0.41±0.03	2.81±0.01	343.00±4.68
LE5-CO4	0.38±0.04	2.89±0.02	350.21±8.65
LE5-CO5	0.36±0.03	2.85±0.01	348.12±5.16
LE6	0.42±0.05	3.27±0.02	546.79±6.90
LE6-CO1	0.41±0.06	3.27±0.02	548.53±7.16
LE6-CO2	0.34±0.03	3.25±0.01	544.16±7.30
LE6-CO3	0.64±0.20	3.42±0.09	560.05±10.14
LE6-CO4	0.38±0.04	3.24±0.01	530.58±6.95
LE6-CO5	0.36±0.06	3.24±0.01	532.00±6.30
LE7	0.26±0.04	1.63±0.01	104.02±2.29
LE7-CO1	0.27±0.03	1.65±0.02	104.94±2.40
LE7-CO2	0.24±0.03	1.66±0.01	103.64±2.09
LE7-CO3	0.27±0.04	1.66±0.03	104.12±2.87
LE7-CO4	0.28±0.04	1.67±0.02	107.33±2.68
LE7-CO5	0.29±0.03	1.63±0.02	103.93±2.16
LE8	1.92±0.35	2.74±0.19	228.74±7.33
LE8-CO1	0.77±0.10	2.58±0.02	217.54±4.39
LE8-CO2	1.13±0.46	2.88±0.08	229.36±3.86
LE8-CO3	1.75±0.42	2.66±0.11	220.50±5.81
LE8-CO4	1.76±0.43	2.88±0.23	229.66±4.85
LE8-CO5	1.49±0.70	2.86±0.33	223.38±6.65
LE9	1.12±0.09	2.91±0.07	336.57±15.95
LE9-CO1	0.93±0.07	2.85±0.04	331.73±9.93
LE9-CO2	1.06±0.09	2.83±0.04	328.92±10.32
LE9-CO3	1.06±0.12	2.78±0.06	333.52±21.72

Table S2 continued from previous page

LE9-CO4	0.76±0.07	2.81±0.04	345.85±9.48
LE9-CO5	1.20±0.14	2.97±0.06	340.21±16.02
LE10	0.25±0.04	1.97±0.02	157.31±2.65
LE10-CO1	0.31±0.05	2.01±0.02	163.07±3.09
LE10-CO2	0.30±0.07	1.98±0.03	158.15±3.24
LE10-CO3	0.28±0.05	1.97±0.03	156.99±3.34
LE10-CO4	0.32±0.08	2.00±0.03	160.48±3.28
LE10-CO5	0.38±0.07	2.02±0.02	161.05±2.61
LE11	0.48±0.05	2.11±0.03	172.44±5.12
LE11-CO1	0.48±0.07	2.09±0.02	173.07±3.91
LE11-CO2	0.43±0.07	2.10±0.02	175.27±3.30
LE11-CO3	0.41±0.06	2.10±0.02	170.30±3.03
LE11-CO4	0.40±0.05	2.12±0.02	173.80±2.87
LE11-CO5	0.41±0.07	2.10±0.02	170.57±2.96

Table S3. Average value of the binding free energy determined by the MM/PBSA method.

System	$\Delta E_{VDWAALS}$	ΔE_{EL}	ΔE_{PB}	ΔE_{NPOLAR}	ΔG_{GAS}	ΔG_{SOLV}	ΔG_{bind}
LE1-CO1	-12.15±10.09	-4.88±6.57	13.00±11.71	-9.90±8.67	-17.03±14.00	20.49±16.58	3.46±4.03
LE1-CO2	-8.69±7.66	-2.31±3.62	6.82±6.87	-6.68±6.40	-11.00±9.95	12.53±10.69	1.53±3.38
LE1-CO3	-21.24±5.79	-4.44±6.75	15.67±7.72	-16.82±4.46	-25.68±9.08	26.51±9.37	0.84±3.25
LE1-CO4	-9.30±8.60	-3.78±5.51	8.61±9.19	-6.60±6.51	-13.08±12.88	14.69±13.51	1.61±2.16
LE1-CO5	-12.57±8.47	-5.69±7.03	12.21±9.47	-9.36±6.58	-18.26±13.38	19.90±13.37	1.64±2.78
LE2-CO1	-28.76 ±2.11	-27.61± 5.96	46.23 ±8.93	-19.80 ±0.91	-56.37± 5.99	65.54± 9.14	9.17± 8.74
LE2-CO2	-26.70± 2.50	-10.27± 6.63	22.59±12.21	-18.91± 1.91	-36.97±7.77	43.05± 12.21	6.08±9.20
LE2-CO3	-23.37±1.82	-4.83±2.33	19.17±10.4	-2.95±0.14	-28.2±3.23	16.22±10.36	-11.98±9.4
LE2-CO4	-25.76± 2.16	-16.21± 6.70	30.18±12.90	-20.00± 1.71	-41.97± 7.46	49.69± 12.97	7.71±9.29
LE2-CO5	-28.81±3.59	-19.68 ± 3.52	39.18± 9.64	-21.41 ±1.19	-48.48± 5.06	59.79± 9.81	11.31±9.67
LE3-CO1	-26.50±3.71	-21.77±4.50	29.32±4.06	-21.10±2.41	-48.27±5.40	44.02±4.47	-4.26±3.64
LE3-CO2	-19.21±8.31	-3.30±5.08	14.48±8.42	-14.89±6.53	-22.51±10.76	26.52±12.28	4.01±4.07
LE3-CO3	-31.48±2.88	-27.71±4.77	41.53±4.95	-23.10±1.28	-59.19±4.68	60.55±5.15	1.36±4.24
LE3-CO4	-22.86±5.30	-7.84±10.42	20.55±12.83	-18.54±4.16	-30.70±13.89	34.10±15.13	3.39±4.09
LE3-CO5	-23.39±2.79	-0.93±4.37	12.42±4.55	-17.74±1.91	-24.32±4.63	25.99±4.77	1.66±3.20
LE4-CO1	-6.14±7.01	-11.92±14.78	13.06±15.4	-5.14±6.46	-18.05±21.38	16.83±18.43	-1.23±4.14
LE4-CO2	-20.1±3.06	-1.92±5.51	12.24±6.52	-15.63±2.46	-22.02±6.6	25.48±7.71	3.46±3
LE4-CO3	-25.82±2.48	-7.63±2.98	24.92±4.77	-19.14±1.61	-33.45±3.89	40.5±5.07	7.05±4.33
LE4-CO4	-0.39±1.57	-0.23±1.54	0.42±2.08	0.23±1.56	-0.62±2.58	1.39±2.94	0.77±0.79
LE4-CO5	-28.65±2.23	-4.4±2.5	16.99±3.75	-20.53±1.27	-33.05±3.69	33.99±4.46	0.94±3.74
LE5-CO1	-32.3±2.33	-25.88±5.43	50.12±5.38	-23.07±1	-58.18±6.02	67.68±5.39	9.51±4.18
LE5-CO2	-3.76±5.54	-0.27±2.31	2.48±4.61	-2.52±4.57	-4.03±6.34	5.61±8.05	1.58±2.42
LE5-CO3	-0.11±0.39	0.05±1.72	-0.02±2.63	0.5±0.39	-0.07±1.94	0.71±2.78	0.64±1.93
LE5-CO4	-29.25±2.54	-12.51±4.94	27.23±4.48	-21.65±1.23	-41.75±5.67	41.57±4.61	-0.18±3.36
LE5-CO5	-12.29±4.04	-10.8±9.56	14.66±8.75	-10.24±3.17	-23.09±12.43	21.53±10.21	-1.56±3.65
LE6-CO1	-17.68±4.26	-16.57±13.47	28.85±12.63	-13.58±3.04	-34.24±13.90	42.22±13.59	7.98±4.42
LE6-CO2	-12.85±6.11	-2.36±4.77	8.76±5.82	-9.82±4.77	-15.21±8.40	16.14±7.82	0.93±2.26
LE6-CO3	-16.06±5.91	-2.02±3.23	11.36±5.66	-12.79±4.83	-18.08±7.19	21.82±8.10	3.74±3.18
LE6-CO4	-29.21±2.60	-9.81±4.53	26.88±4.64	-21.47±1.61	-39.02±5.31	44.80±5.31	5.78±4.33
LE6-CO5	-16.75±4.68	-1.02±5.45	9.59±5.88	-13.76±3.52	-17.77±6.92	20.60±6.99	2.83±2.68
LE7-CO1	-9.91±6.34	-2.66±7.53	7.53±8.25	-7.71±5.27	-12.57±10.18	13.17±10.06	0.6±2.29
LE7-CO2	-13.79±3.98	-1.18±5.25	6.99±5.41	-10.41±2.61	-14.97±6.62	14.96±6.01	-0.01±1.91
LE7-CO3	-3.52±5.21	0.08±3.59	2.04±4.67	-2.27±4.07	-3.44±6.38	4.43±6.21	0.99±1.36
LE7-CO4	-0.32±1.25	-1.02±5.15	1.41±5.48	0.34±1.05	-1.34±5.63	2.33±6	0.99±0.96
LE7-CO5	-7.14±8.3	-2.01±6.98	5.5±9.59	-5.11±6.56	-9.15±13.35	9.76±12.7	0.62±1.84
LE8-CO1	-0.50±1.93	-0.19±2.29	0.47±2.70	0.14±1.71	-0.70±3.31	1.40±3.46	0.71±0.70
LE8-CO2	-22.19±7.50	-3.07±4.64	13.22±6.45	-17.66±5.91	-25.26±9.90	24.48±8.87	-0.78±3.12
LE8-CO3	-11.68±6.34	-2.88±5.08	9.05 ±7.70	-9.01±5.06	-14.57±9.35	16.39±10.64	1.82±3.41
LE8-CO4	-21.59±5.42	-17.67±12.46	26.47±11.88	-17.60±4.15	-39.26±15.13	37.74±13.34	-1.53±3.56
LE8-CO5	-0.75±2.43	-0.45±3.68	0.87±4.28	-0.11±2.34	-1.20±5.04	1.98±5.34	0.78±0.84
LE9-CO1	-33.20±2.19	-28.73±5.37	55.31±6.65	-23.24±1.09	-61.93±5.90	72.76±6.72	10.72±5.52
LE9-CO2	-6.94±6.58	-1.36±3.78	4.73±5.32	-5.03±5.17	-8.30±8.37	9.26±8.23	0.96±1.75
LE9-CO3	-24.24±2.55	-13.23±3.84	21.66±3.27	-20.37±1.47	-37.47±5.01	34.23±3.49	-3.24±3.06
LE9-CO4	-27.25±2.72	-24.82±10.07	40.06±8.79	-22.10±1.61	-52.06±10.55	55.10±8.95	3.04±4.98
LE9-CO5	-21.24±5.06	-10.09±9.22	22.08±9.40	-16.82±3.60	-31.33±10.99	34.88±10.79	3.56±3.43
LE10-CO1	-31.32±2.24	-17.38±4.1	33.49±2.83	-23.58±0.91	-48.7±4.16	47.84±2.91	-0.85±3.47
LE10-CO2	-32.79±1.89	-15.74±3.38	31±2.82	-22.8±0.71	-48.53±3.32	49.29±2.83	0.76±3.15
LE10-CO3	-30.79±2.44	-4.05±2.21	24.41±3.54	-23.85±1.46	-34.83±3.83	39.98±3.74	5.15±3.01
LE10-CO4	-14.34±4.94	-3.49±4.39	10.72±5.91	-11.32±3.75	-17.83±7.47	19.77±7.66	1.94±3.27
LE10-CO5	-18.95±3.16	-5.67±4.02	14.05±3.45	-15.5±2.41	-24.62±5.5	24.22±4.13	-0.39±2.87
LE11-CO1	-24.53±4.35	-23.20±13.64	35.92±14.94	-20.46±2.98	-47.73±16.64	50.10±16.15	2.36±4.48
LE11-CO2	-30.77±2.91	-8.21±4.48	22.02±5.18	-22.66±1.64	-38.98±5.29	38.80±6.20	-0.18±4.41
LE11-CO3	-22.57±3.94	-2.31±3.85	12.06±5.22	-18.45±2.23	-24.88±6.68	25.32±6.46	0.43±3.26
LE11-CO4	-9.30±10.33	-5.99±8.69	10.15±12.13	-7.04±8.22	-15.29±17.34	15.82±17.01	0.52±3.09
LE11-CO5	-20.36±3.47	-1.97±4.14	10.77±5.48	-17.79±2.67	-22.33±5.28	23.91±6.37	1.58±3.12

$E_{VDWAALS}$ = Van der Waals energy; E_{EL} = Electrostatic energy; E_{PB} = Electrostatic contribution free energy calculated by Poisson-Boltzmann; E_{NPOLAR} : Non-polar solvation energy; ΔG_{gas} = Estimated binding free energy phase gas; ΔG_{solv} = Estimates binding free energy solvent; ΔG_{bind} = Estimated binding free energy. All values are in kcal/mol.

Table S4. Average value of the binding free energy determined by the MM/GBSA method.

System	$\Delta E_{VDWAALS}$	ΔE_{EL}	ΔE_{GB}	ΔE_{SURF}	ΔG_{GAS}	ΔG_{SOLV}	ΔG_{bind}
LE1-CO1	-15.35±6.05	-9.04±8.39	18.56±9.45	-2.2±1.03	-24.39±11.79	16.36±8.77	-8.03±3.89
LE1-CO2	-6.68±7.32	-1.44±3.13	5.12±5.79	-0.98±1.1	-8.12±9.15	4.14±4.85	-3.98±4.97
LE1-CO3	-14.71±8.8	-6.93±7.51	14.15±10.22	-2.21±1.34	-21.64±14.67	11.93±9.06	-9.7±6.39
LE1-CO4	-16.06±3.5	-5.23±6.13	13.68±6.37	-2.14±0.44	-21.29±7.04	11.54±6.17	-9.75±2.71
LE1-CO5	-8.24±9.09	-0.93±6.18	5.41±7.65	-1.27±1.39	-9.17±11.21	4.14±6.86	-5.03±6.02
LE2-CO1	-28.56±1.84	-24.4±4.56	37.48±2.93	-3.66±0.17	-52.97±4.46	33.82±2.9	-19.15±2.42
LE2-CO2	-24.95±2.49	-6.34±3.19	17.64±4.06	-3±0.38	-31.29±5.07	14.64±3.75	-16.66±2.06
LE2-CO3	-23.97±1.76	-4.44±1.81	14.37±1.52	-2.93±0.2	-28.41±2.6	11.44±1.48	-16.97±1.82
LE2-CO4	-27.09±1.72	-20.87±4.49	30.37±2.72	-3.91±0.23	-47.96±4.85	26.46±2.61	-21.5±2.86
LE2-CO5	-25.74±1.72	-19.01±3.61	27.22±2.21	-3.74±0.2	-44.75±3.26	23.48±2.16	-21.27±2
LE3-CO1	-23.62±3.78	-22.20±4.51	25.46±3.61	-3.39±0.43	-45.82±5.11	22.07±3.52	-23.75±3.58
LE3-CO2	-6.10±7.30	-1.44±5.30	5.47±7.53	-0.88±1.04	-7.54±10.26	4.60±6.69	-2.95±4.10
LE3-CO3	-32.79±2.55	-26.62±3.44	32.59±2.15	-4.53±0.19	-59.41±3.80	28.06±2.14	-31.35±3.06
LE3-CO4	-18.50±4.64	0.24±4.02	9.31±3.75	-2.70±0.85	-18.26±4.53	6.61±3.70	-11.64±3.30
LE3-CO5	-25.62±2.19	1.05±4.02	14.12±3.72	-3.39±0.21	-24.57±4.58	10.73±3.68	-13.84±2.15
LE4-CO1	-6.14±7.01	-11.92±14.78	13.52±15.2	-0.99±1.11	-18.05±21.38	12.54±14.13	-5.51±7.83
LE4-CO2	-20.1±3.06	-1.92±5.51	12.86±5.53	-2.82±0.44	-22.02±6.6	10.03±5.34	-11.99±2.83
LE4-CO3	-25.82±2.48	-7.63±2.98	21.43±2.79	-3.6±0.38	-33.45±3.89	17.83±2.61	-15.63±2.42
LE4-CO4	-0.39±1.57	-0.23±1.54	0.67±2.28	-0.05±0.25	-0.62±2.58	0.62±2.08	0±0.74
LE4-CO5	-28.65±2.23	-4.4±2.5	18.68±2.61	-3.51±0.23	-33.05±3.69	15.17±2.6	-17.88±2.01
LE5-CO1	-32.3±2.33	-25.88±5.43	39.17±3.78	-4.16±0.2	-58.18±6.02	35.02±3.68	-23.16±3.33
LE5-CO2	-3.76±5.54	-0.27±2.31	2.64±4.41	-0.48±0.71	-4.03±6.34	2.16±3.83	-1.88±2.89
LE5-CO3	-0.11±0.39	0.05±1.72	0.14±1.89	-0.01±0.06	-0.07±1.94	0.13±1.86	0.06±0.25
LE5-CO4	-29.25±2.54	-12.51±4.94	23.65±3.68	-3.98±0.22	-41.75±5.67	19.67±3.61	-22.08±3.01
LE5-CO5	-12.29±4.04	-10.8±9.56	14.29±8.31	-1.77±0.51	-23.09±12.43	12.53±7.96	-10.57±4.97
LE6-CO1	-13.38±2.86	-11.58±7.04	23.06±6.07	-1.9±0.45	-24.96±7.85	21.16±5.84	-3.8±2.86
LE6-CO2	-12.51±6.02	-1.94±4.03	7.8±4.99	-1.66±0.81	-14.45±7.52	6.14±4.56	-8.31±4.49
LE6-CO3	-15.8±3.75	-2.24±4.09	11.59±4.45	-2.3±0.63	-18.04±6.68	9.29±4.03	-8.75±3.45
LE6-CO4	-28.98±2.13	-7.86±4.08	21.83±2.81	-3.73±0.24	-36.84±4.83	18.1±2.7	-18.74±2.72
LE6-CO5	-21.98±4.36	-1.65±7.37	15.67±7.41	-3.04±0.57	-23.63±8.19	12.64±7.36	-10.99±3.35
LE7-CO1	-9.91±6.34	-2.66±7.53	8.09±8.11	-1.41±0.91	-12.57±10.18	6.68±7.73	-5.89±4.46
LE7-CO2	-13.79±3.98	-1.18±5.25	8.02±5.32	-1.87±0.49	-14.97±6.62	6.15±5.24	-8.82±3.35
LE7-CO3	-3.52±5.21	0.08±3.59	1.91±4.43	-0.48±0.68	-3.44±6.38	1.44±4.09	-2.01±3.57
LE7-CO4	-0.32±1.25	-1.02±5.15	1.49±5.6	-0.04±0.18	-1.34±5.63	1.45±5.53	0.12±0.52
LE7-CO5	-7.14±8.3	-2.01±6.98	5.56±9.48	-1.02±1.18	-9.15±13.35	4.54±8.59	-4.6±5.91
LE8-CO1	-0.11±0.41	-0.35±2.38	0.59±2.5	-0.02±0.09	-0.45±2.54	0.58±2.42	0.12±0.37
LE8-CO2	-11.87±9.44	-1.29±4.89	7.85±6.47	-1.74±1.41	-13.16±10.42	6.1±5.55	-7.06±6.25
LE8-CO3	-1.32±3.22	-0.33±1.95	1.25±3.17	-0.2±0.48	-1.65±4.45	1.05±2.76	-0.61±1.96
LE8-CO4	-25.85±2.48	-10.46±4.1	20.69±4.28	-3.7±0.33	-36.31±5.43	16.99±4.11	-19.32±2.25
LE8-CO5	-0.69±2.09	-0.39±4.25	0.99±4.2	-0.1±0.33	-1.07±4.7	0.9±4.1	-0.17±1.14
LE9-CO1	-32.98±2.01	-28.55±4.67	43.02±3.97	-4.24±0.17	-61.53±4.92	38.78±3.93	-22.75±2
LE9-CO2	-2.81±4.05	-0.73±2.69	2.47±3.95	-0.4±0.58	-3.54±5.76	2.06±3.49	-1.48±2.69
LE9-CO3	-25.28±2.66	-15.09±2.71	24.58±2.72	-3.82±0.27	-40.37±3.91	20.76±2.57	-19.61±2.08
LE9-CO4	-26.83±3.3	-27.06±11.9	38.81±9.37	-4.04±0.43	-53.88±13.08	34.77±9.14	-19.12±4.55
LE9-CO5	-19.92±3.47	-14.25±11.93	24.54±9.91	-3.09±0.41	-34.17±11.4	21.45±9.9	-12.73±3.16
LE10-CO1	-31.32±2.24	-17.38±4.1	28.53±2.64	-4.52±0.15	-48.7±4.16	24.01±2.59	-24.68±2.68
LE10-CO2	-32.79±1.89	-15.74±3.38	23.96±2	-4.36±0.13	-48.53±3.32	19.6±2.01	-28.93±2.36
LE10-CO3	-30.79±2.44	-4.05±2.21	17.17±2.39	-4.71±0.3	-34.83±3.83	12.46±2.21	-22.37±2.53
LE10-CO4	-14.34±4.94	-3.49±4.39	10.96±4.91	-2.02±0.73	-17.83±7.47	8.95±4.39	-8.88±3.95
LE10-CO5	-18.95±3.16	-5.67±4.02	13.35±3.08	-2.76±0.44	-24.62±5.5	10.59±2.9	-14.03±3.42
LE11-CO1	-27.1±2.49	-30.99±4.99	39.91±3.29	-4.15±0.18	-58.09±4.52	35.76±3.25	-22.33±2.47
LE11-CO2	-32.19±1.89	-10.39±2.98	22.29±2.37	-4.3±0.22	-42.58±3.8	17.99±2.25	-24.6±2.28
LE11-CO3	-24.11±2.16	-5.07±2.88	14.41±1.91	-3.65±0.23	-29.18±3.92	10.76±1.86	-18.42±2.88
LE11-CO4	-23.48±5.45	-9.12±3.31	17.45±3.15	-3.34±0.76	-32.6±7.16	14.11±2.78	-18.48±5.55
LE11-CO5	-22.3±3.34	0.94±2.61	10.86±3.29	-3.22±0.54	-21.36±4.67	7.64±3	-13.73±2.39

$E_{VDWAALS}$ = Van der Waals energy; E_{EL} = Electrostatic energy; E_{GB} = Electrostatic contribution free energy calculated by Generalized Born; E_{SURF} = Non-polar contribution calculated based on the solvent-accessible surface area; ΔG_{gas} = Estimated binding free energy phase gas; ΔG_{solv} = estimates binding free energy solvent; ΔG_{bind} = Estimated binding free energy. All values are in kcal/mol.
